# Dexmedetomidine improves the outcomes for pediatric severe sepsis with mechanical ventilation

**DOI:** 10.1186/s12887-023-04232-6

**Published:** 2023-08-18

**Authors:** Chun Zhao, Yi Yin, Tianxin Zhang, Jing Li, Xiaoming Zhou, Yujuan Wang, Wei Wang, Qiwei Wang, Youpeng Jin

**Affiliations:** 1 Department of Pediatric intensive care unit, Shandong Provincial Hospital Affiliated to Shandong First Medical University, Jinan, Shandong Province P.R. China; 2 Department of Biostatistics, School of Public Health, Cheeloo College of Medicine, Shandong University, Jinan, Shandong Province P.R. China; 3 Department of Pediatrics, Qingdao Women and Children Hospital, Qingdao, Shandong Province P.R. China; 4 Scientific research department, Shandong Provincial Hospital Affiliated to Shandong First Medical University, Jinan, Shandong Province P.R. China

**Keywords:** Dexmedetomidine, Pediatric, Outcome, Severe sepsis, Mechanical ventilation

## Abstract

**Background:**

The sedative dexmedetomidine has been shown to reduce mortality in adult patients with severe sepsis, but it is not known whether children benefit. This study explored the effects of dexmedetomidine on the outcomes of children with severe sepsis with mechanical ventilation.

**Methods:**

In this retrospective cohort study, children with severe sepsis requiring mechanical ventilation from 2016 to 2020 were categorized as dexmedetomidine and non-dexmedetomidine group. The propensity score matching was performed to match cases in both groups. The primary outcome was 28-day mortality, and the secondary outcomes were acute kidney injury, ventilator-free days, lengths of PICU and hospital stays. The Kaplan-Meier method and was the log-rank test used to estimate the 28-day mortality rate and assess between-group differences.

**Results:**

In total, 250 patients were eligible patients: 138 in the dexmedetomidine group and 112 in the non-dexmedetomidine group. After 1:1 propensity score matching, 61 children in each group. dexmedetomidine group showed more lower 28-day mortality (9.84% vs. 26.23%, P = 0.008). During the 7-day observation period after PICU admission, the dexmedetomidine group showed significantly lower neurological and renal sub-scores at day 7 and serum creatinine level at day 3 and day 7. There were no statistical differences in the incidence of acute kidney injury, ventilator-free days, lengths of PICU and hospital stays between the two groups.

**Conclusions:**

dexmedetomidine treatment in children with severe sepsis is associated with better outcomes and should therefore be considered for the sedation strategy.

**Supplementary Information:**

The online version contains supplementary material available at 10.1186/s12887-023-04232-6.

## Introduction

Sepsis is a serious inflammatory condition that can develop after dysregulation of the host immune response to infection [1]. Proper care of patients with sepsis, especially those requiring mechanical ventilation, often requires sedation to reduce the stress and anxiety associated with invasive interventions [1–5]. Dexmedetomidine is a selective α2-adrenergic agonist that is administered as a sedative, [6] and has been shown to attenuate inflammatory reactions and organ damage in both animal and adult studies [7–10]. Notably, some studies have shown that the use of dexmedetomidine is associated with reduced mortality among adult patients with sepsis [9, 11].

The use of dexmedetomidine in infants and children in Europe and the United States has been increasing for several years [12]. In China, dexmedetomidine is recommended in the experts’ consensus on analgesia and sedation for children in pediatric intensive care unit (PICU) [13]. Due to the particularity of children, the pharmacokinetics of dexmedetomidine in children is different from that in adults [14]. The effects of dexmedetomidine on the mortality of pediatric patients with sepsis have not been reported, even though sepsis is among the leading causes of death among children worldwide [2]. Therefore, we conducted a retrospective cohort study to evaluate the effects of dexmedetomidine on the outcome of mechanically ventilated children with severe sepsis in Shandong Province, China.

## Methods

### Study design and patient selection

This multicenter retrospective cohort study was conducted in the PICUs of two tertiary care hospitals in Shandong Province, China: Shandong provincial Hospital Affiliated to Shandong First Medical University and Qingdao Women and Children Hospital. Data from children admitted to these centers with severe sepsis requiring mechanical ventilation between 1 and 2016 and 31 December 2020 were screened for inclusion in the study. The information of the children was obtained through the electronic medical record inquiry system. The study protocol conformed to the ethical guidelines of the 1975 Declaration of Helsinki and has been registered in the Chinese Clinical Trial Registration Center (ID: ChiCTR2100047250) and approved by the local ethics committee of each participating hospital (SWYX. NO: 2021 − 187). Guardian consent was waived for this type of study.

Researchers were trained before data collection. During the study, the coordinator and physicians in charge were responsible for validating the collected data and checking for any suspicious errors or missing values.

**Fig. 1:**
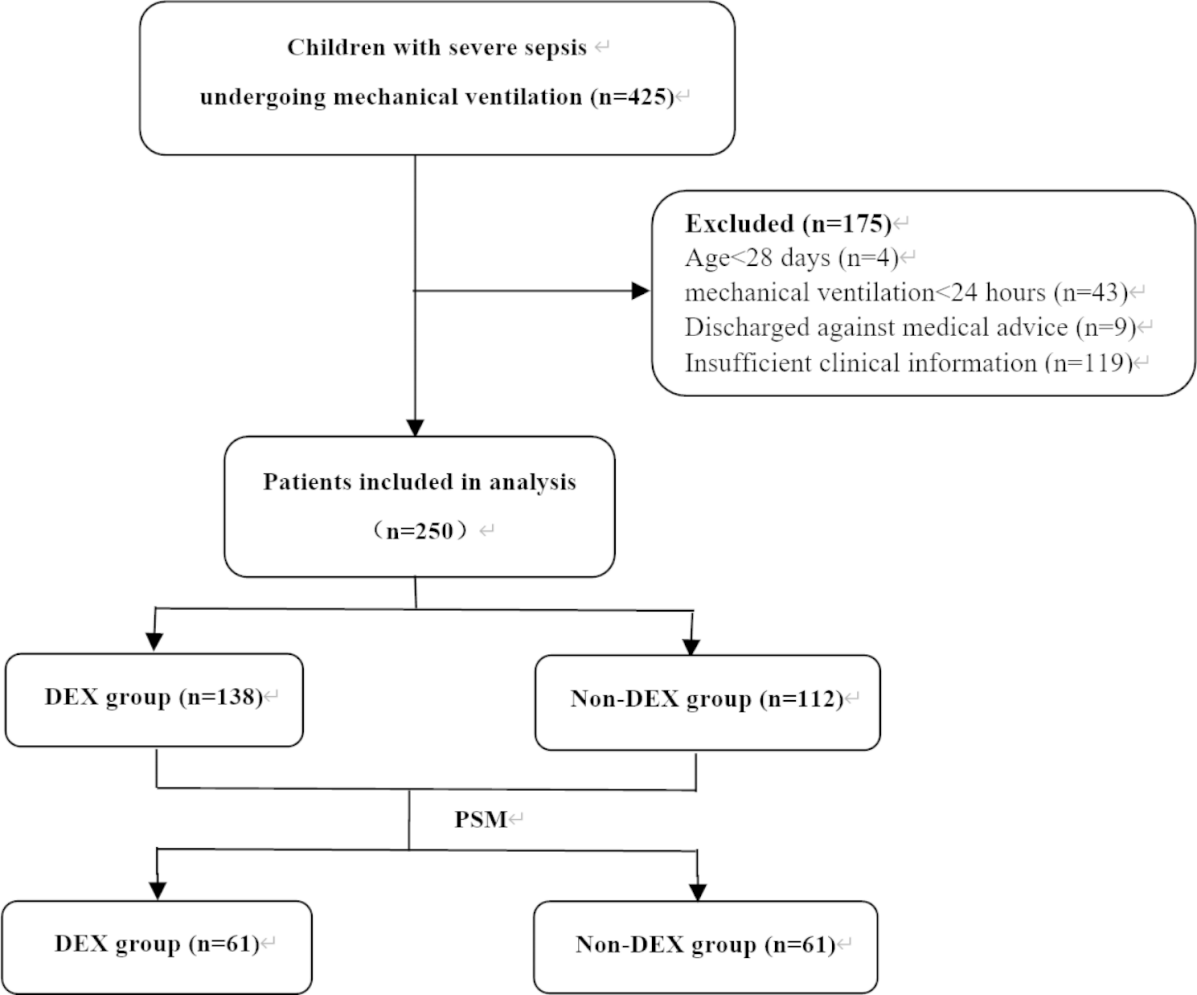
Flow diagram of study population selection. Abbreviations: PSM, propensity score matching

The criteria for inclusion were (i) an age between 28 days and 18 years, (ii) patients with severe sepsis diagnosed according to the International Pediatric Sepsis Consensus Conference: definitions for sepsis and organ dysfunction in pediatrics, [15] (iii) patients undergoing mechanical ventilation for at least 24 h. Patients were excluded from the study if they were discharged against medical advice, or if there was insufficient clinical information. Patients who met the inclusion criteria were divided into DEX group (treated with dexmedetomidine) and non-DEX group (treated without dexmedetomidine) on the basis of the sedation strategy during PICU hospitalization. (Fig. 1).

### Comprehensive medical treatment

All patients were administered standardized treatment according to the International Pediatric Sepsis Consensus Conference (2005) and Surviving Sepsis Campaign International Guidelines (2012) [15, 16].

### Sedative and analgesia strategy

**Fig. 2:**
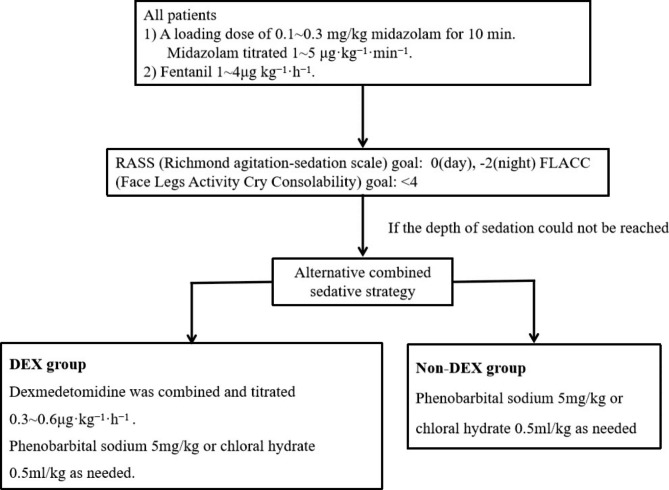
Sedation & analgesia protocol. Abbreviations: RASS, Richmond agitation-sedation scale; FLACC, Face Legs Activity Cry Consolability; DEX, dexmedetomidine

All patients received midazolam for sedation and fentanyl for analgesia. The loading dose of midazolam 0.1–0.3 mg/kg body weight was intravenous infusion for 10 min, followed by a continuous intravenous infusion at 1–5 µg·kg^− 1^·min^− 1^, and fentanyl titrated at 1 ~ 4 µg·kg^− 1^·h^− 1^.The aim of sedation was to establish a score from − 2 to 0 on the Richmond agitation sedation scale (RASS). The goal of analgesia was Face Legs Activity Cry Consolability (FLACC) < 4. If the depth of sedation could not be reached, other sedative drugs (dexmedetomidine or phenobarbital sodium or chloral hydrate) will be combined. Sedation was maintained throughout the duration of mechanical ventilation or as needed. According to whether dexmedetomidine was used, patients were separated into two groups including the DEX group (received dexmedetomidine 0.3 ~ 0.6 µg·kg^− 1^·h^− 1^ intravenous infusion as combined sedation) and non-DEX group (received phenobarbital sodium or chloral hydrate as combined sedation) (Fig. 2).The amount and the duration of dexmedetomidine used see in Suppl Table 1.

### Definitions

Sepsis-induced acute kidney injury (AKI) is defined as any of the following (not graded): increase in serum creatinine level (Scr) by ≥ 0.3 mg/dl (≥ 26.5 µmol/l) within 48 h; or increase in Scr to ≥ 1.5 times baseline, which is known or presumed to have occurred within the prior 7 days; or urine volume < 0.5ml/kg/hour for 6 h [17].

The pediatric Sequential Organ Failure Assessment (pSOFA) score included six sub-scores for six organ systems (respiratory, coagulation, hepatic, cardiovascular, neurologic, and renal). The worst value for every variable in each 24-hour period was used to calculate the sub-score for each of the 6 organ systems. Each sub-score ranges 0–4 points, and higher score indicate a worse outcome. Daily pSOFA score was the sum of the 6 sub-scores [18] (Suppl Table 2).

### Outcomes

The primary outcomes were 28-day mortality. The secondary outcomes were the number of ventilator-free days in a period of 28 days, the lengths of PICU and hospital stays, the incidence of sepsis-induced AKI, and the trajectory of subscores of the pSOFA which were obtained at day 1, 3, and 7 of PICU admission.

### Statistical analysis

For baseline characteristics, quantitative data (non-normally distributed) were expressed as medians and interquartile ranges (IQRs) and compared by the Mann-Whitney U test. Qualitative data were expressed as numbers and percentages and compared by the χ [2] test or Fisher’s exact test.

To balance baseline characteristics between groups and estimate the association between DEX administration and outcomes in children with severe sepsis, propensity score matching (PSM) was performed using 1:1 nearest neighbor matching (caliper value is 0.2) to match cases in both groups. Propensity scores were adjusted for age, weight, ratio of surgical patients, pSOFA-1, number of organ damage, proportion of patients with septic shock, comorbidities, and use of vasoactive drugs, blood purification, as well as fluid resuscitation estimating the probability by logistic regression. The standardized mean difference was calculated to evaluate the efficiency of propensity score matching in reducing the differences between the two groups. To account for missing values and correlations among repeated measurements, the generalized estimating equation(GEE)was adopted for comparisons of pSOFA and biological markers changes between the groups.

For comparison of the probability of patients’ 28-day survival for two groups, the Kaplan–Meier survival method was used to draw survival probability curves and the log–rank test was used for statistical assessment. Cox proportional-hazards analysis was used to assess the relationship between DEX administration and mortality, corrected for baseline variables. The effect of DEX use on the incidence of AKI was estimated using a logistic regression model. Linear regression was used to evaluate the association between DEX use and the ventilator-free days, the length of PICU and hospital stay.

All probability values were two-tailed, and statistical significance was defined as *P* < 0.05. Statistical analysis was performed using R 4.1.1 software for Windows.

## Results

### Patients’ characteristics

The two PICUs, located in Jinan and Qingdao, China, had a mean of 20 beds each, and these two units accepted both medical and surgical patients. A total of 425 children with severe sepsis requiring mechanical ventilation were initially enrolled, and finally 250 met the inclusion criteria: 138 in the DEX group and 112 in the non-DEX group (Fig. 1). The overall 28-day mortality of these 250 pediatric severe sepsis was 18.8%. There were no differences between the DEX group and non-DEX group in sex ratio, hospital acquired infections ratio, types of pathogens, proportion of patients receiving nephrotoxic drugs and glucocorticoid. Patients in non-DEX group were younger than those in the DEX group. The pSOFA-1 day was significantly higher in the DEX group (7.0 vs. 5.50, P = 0.007). The number of organs damaged in the DEX group was significantly more than in the non-DEX group (3 vs. 2, P = 0.013), and more patients in the DEX group complicated with septic shock (48.55% vs. 34.82%, P = 0.040). In addition, more patients in the DEX group received vasoactive drugs, fluid resuscitation and blood purification (P<0.001) (Table 1).

**Table 1:** Demographic and clinical characteristics of pediatric severe sepsis with mechanical ventilation

Characteristic	Total(n = 250)	Non-Dex group(n = 112)	Dex group(n = 138)	P value	SMD/χ^2^
Male sex (n [%])	147 (58.80%)	67 (59.82%)	80 (57.97%)	0.868	0.038
Age, yr (median [IQR])	1.84 (0.60,5.18)	1.13 (0.37,4.09)	2.19 (0.88,6.26)	< 0.001	0.421
Weight (kg) (median [IQR])	11.50 (8.12,19.00)	10.00 (6.22,16.92)	12.95 (10.00,20.90)	< 0.001	0.338
Surgical patients (n [%])	15 (6.00%)	2 (1.79%)	13 (9.42%)	0.024	0.337
^*^pSOFA-1 day	6.00 (4.00,10.00)	5.50 (3.00,8.25)	7.00 (4.00,10.00)	0.007	0.360
No. of damaged organ (median [IQR])	3.00 (2.00,4.00)	2.00 (2.00,3.25)	3.00 (2.00,4.00)	0.013	0.316
Septic shock (n [%])	106 (42.40%)	39 (34.82%)	67 (48.55%)	0.040	0.281
Comorbidities					
Cardiovascular	18 (7.20%)	13 (11.61%)	5 (3.62%)	0.029	0.304
Respiratory	8 (3.20%)	6 (5.36%)	2 (1.45%)	0.145	0.217
Gastrointestinal	15 (6.00%)	8 (7.14%)	7 (5.07%)	0.676	0.087
Renal and Urologic	14 (5.60%)	6 (5.36%)	8 (5.80%)	1	0.019
Neurologic and Neuromuscular	17 (6.80%)	10 (8.93%)	7 (5.07%)	0.341	0.152
Hematologic or Malignancy	27 (10.80%)	7 (6.25%)	20 (14.49%)	0.060	0.273
Metabolic	4 (1.60%)	3 (2.68%)	1 (0.72%)	0.328	0.152
Other Congenital or Genetic Defect	14 (5.60%)	9 (8.04%)	5 (3.62%)	0.218	0.189
Miscellaneous	3 (1.20%)	1 (0.89%)	2 (1.45%)	1	0.052
Hospital acquired infections (n [%])	12 (4.84%)	5 (4.13%)	7 (5.51%)	0.613	0.256
Pathogens (n [%])					
G + bacteria	41 (16.40%)	19 (16.96%)	22 (15.94%)	0.964	0.028
G^−^ bacteria	38 (15.20%)	21 (18.75%)	17 (12.32%)	0.218	0.178
Fungi	9 (3.60%)	5 (4.46%)	4 (2.90%)	0.520	0.083
Others	8 (3.20%)	2 (1.79%)	6 (4.35%)	0.303	0.149
Treatment					
Vasoactive drugs (n [%])	122 (48.80%)	40 (35.71%)	82 (59.42%)	< 0.001	0.489
Blood purification (n [%])	126 (50.40%)	27 (24.11%)	99 (71.74%)	< 0.001	1.085
Fluid resuscitation (n [%])	222 (88.80%)	90 (80.36%)	132 (95.65%)	< 0.001	0.484
Glucocorticoid (n [%])	118 (47.20%)	45 (40.18%)	73 (52.90%)	0.061	0.257
Outcome					
28-day mortality	47(18.8%)	29(25.89%)	18(13.04%)	0.015	0.042

Abbreviations: SMD, standardized mean difference; pSOFA, pediatric Sequential Organ Failure Assessment; G^+^ bacteria, Gram-positive bacteria; G^−^ bacteria, Gram-negative bacteria

*pSOFA-1 day was calculated within the first 24 h after PICU admission using the value associated with the greatest severity of illness

We performed 1:1 PSM to balance the baseline characteristics between the two groups, resulting in 61 cases in each group. After PSM, the baseline characteristics were balanced between the two groups, such as age, weight, ratio of surgical patients, pSOFA-1 day, number of organ damage, proportion of patients with septic shock, comorbidities, and use of vasoactive drugs, blood purification, as well as fluid resuscitation (Table 2).

**Table 2:** Comparisons of the covariates after propensity score matching

Characteristic	Non-Dex group (n = 61)	Dex group (n = 61)	P	SMD/χ^2^
Male sex (n [%])	32 (52.46%)	37 (60.66%)	0.465	0.166
Age, yr (median [IQR])	1.54 (0.54,5.24)	1.99 (0.79,4.86)	0.432	0.009
Weight (kg) (median [IQR])	11.00 (8.12,23.00)	11.50 (9.00,18.50)	0.838	0.073
Surgical patients (n [%])	1 (1.64%)	5 (8.20%)	0.207	0.307
pSOFA-1 day	6.00 (4.00,10.00)	6.00 (4.00,8.00)	0.572	0.085
No. of organs damaged (median [IQR])	3.00 (2.00,4.00)	3.00 (2.00,3.00)	0.671	0.064
Septic Shock (n [%])	22 (36.07%)	26 (42.62%)	0.578	0.135
Comorbidities				
Cardiovascular	6 (9.84%)	3 (4.92%)	0.491	0.189
Respiratory	4 (6.56%)	0 (0.00%)	0.119	0.375
Gastrointestinal	4 (6.56%)	5 (8.20%)	1	0.063
Renal and Urologic	2 (3.28%)	3 (4.92%)	1	0.083
Neurologic and Neuromuscular	5 (8.20%)	2 (3.28%)	0.439	0.213
Hematologic or Malignancy	2 (3.28%)	9 (14.75%)	0.058	0.409
Metabolic	0 (0.00%)	1 (1.64%)	1	0.183
Other Congenital or Genetic Defect	4 (6.56%)	1 (1.64%)	0.365	0.250
Miscellaneous	0 (0.00%)	1 (1.64%)	1	0.183
Pathogens (n [%])				
G^+^ bacteria	10 (16.39%)	11 (18.03%)	1	0.043
G^−^bacteria	11 (18.03%)	5 (8.20%)	0.18	0.295
Fungi	2 (3.28%)	2 (3.28%)	1	0.001
Others	2 (3.28%)	4 (6.56%)	0.68	0.152
Treatment				
Vasoactive drugs (n [%])	30 (49.18%)	31 (50.82%)	1	0.033
Blood purification (n [%])	27 (44.26%)	30 (49.18%)	0.717	0.099
Fluid resuscitation (n [%])	56 (91.80%)	56 (91.80%)	1	0.001
Glucocorticoid (n [%])	35 (57.38%)	26 (42.62%)	0.147	0.298

### Effects of dexmedetomidine on laboratory values and pSOFA scores over the 7-day observation period

Urine output volumes and Scr, bood urea nitrogen (BUN), Interleukin-6 (IL-6), procalcitonin (PCT) and lactic acid (Lac) levels on day 1, 3, and 7 after PICU admission are shown in Table 3. After PSM, compared the changes in the non-DEX group from baseline to day 7, the DEX group showed more favorable changes in Scr level at day 3 and day 7. However, there were no significant differences between the two group in the changes of IL-6, PCT, BUN, Lac and urine output volumes. (Table 3)

**Table 3:** Effects of DEX on laboratory values and pSOFA scores of mechanically ventilated pediatric severe sepsis over a 7-day observation period

	Non-Dex group(n = 61)	Dex group(n = 61)	Beta(95%CI)	P
IL-6				
Day 1	51.40 (4.30, 556.78)	80.15 (6.52, 810.00)		
Day 3	17.54 (6.78, 69.16)	26.41 (7.13, 88.15)	-9.06 (-22.17,12.44)	0.350
Day 7	6.08 (2.77, 18.57)^*†^	10.05 (1.92, 22.60) ^*^	-4.19 (-19.11,12.04)	0.701
BUN				
Day 1	4.60 (3.40,8.50)	4.60 (3.00,8.10)		
Day 3	4.05 (2.68,7.68)	4.20 (2.90,7.10)	-0.80 (-2.58,0.97)	0.376
Day 7	4.85 (2.50,5.85)	3.95 (2.82,6.75)	-0.85 (-2.28,0.58)	0.245
Scr				
Day 1	32.66 (20.89,63.52)	30.55 (21.39,41.16)		
Day 3	29.23 (18.26,70.03)	30.44 (19.00,50.40)	-20.65 (-40.74,-0.56)	0.044
Day 7	23.00 (17.25,58.53)	23.99 (17.14,33.75) ^*^	-19.47 (-35.1,-3.83)	0.015
Urine output				
Day 1	2.36 (1.12,3.73)	2.22 (1.40,3.61)		
Day 3	3.20 (1.98,4.73)	3.29 (2.50,4.20)	-0.23 (-0.77,0.31)	0.395
Day 7	3.47 (2.18,4.77)	3.31 (2.46,4.97) ^*^	-0.17 (-0.62,0.27)	0.446
Lac				
Day 1	1.30 (0.90,2.00)	1.10 (0.80,1.70)		
Day 3	1.40 (1.00,2.30)	1.00 (0.70,1.60)	-0.56 (-1.22,0.09)	0.093
Day 7	0.80 (0.50,1.90)	0.90 (0.60,1.60)	-0.51 (-1.16,0.13)	0.120
pSOFA				
Day 1	6.00 (4.00,10.00)	6.00 (4.00,8.00)		
Day 3	6.00 (3.00,10.00)	6.00 (4.00,9.00)	-0.1(-1.14,0.93)	0.845
Day 7	4.00 (2.00,6.00) ^*†^	3.00 (2.00,7.00) ^*†^	-0.08(-0.94,0.78)	0.859
Respiratory subscore				
Day 1	2.50 (1.00,3.75)	2.00 (0.00,3.00)		
Day 3	2.00 (0.00,3.00)	2.00 (0.00,3.00)	-0.31(-0.74,0.11)	0.148
Day 7	1.00 (0.00,2.75)	1.00 (0.00,2.00)	-0.27(-0.63,0.09)	0.137
Coagulation subscore				
Day 1	0.00 (0.00,2.00)	0.00 (0.00,2.00)		
Day 3	1.00 (0.00,2.00)	0.00 (0.00,2.00)	-0.21(-0.53,0.11)	0.192
Day 7	0.00 (0.00,1.00) ^†^	0.00 (0.00,1.50)	-0.15(-0.41,0.12)	0.287
Hepatic subscore				
Day 1	0.00 (0.00,0.00)	0.00 (0.00,0.00)		
Day 3	0.00 (0.00,0.25)	0.00 (0.00,0.00)	0(-0.23,0.23)	0.989
Day 7	0.00 (0.00,0.00)	0.00 (0.00,0.00)	-0.05(-0.24,0.15)	0.644
Cardiovascular subscore				
Day 1	0.00 (0.00,3.00)	1.00 (0.00,1.00)		
Day 3	1.00 (0.00,3.00)	1.00 (0.00,3.00)	0.04(-0.33,0.4)	0.849
Day 7	0.00 (0.00,1.00)	1.00 (0.00,1.00)	0.09(-0.21,0.4)	0.542
Neurological subscore				
Day 1	2.50 (1.00,3.00)	2.00 (1.00,3.00)		
Day 3	3.00 (1.00,3.00)	2.00 (1.00,3.00)	-0.3(-0.63,0.03)	0.075
Day 7	2.00 (1.00,3.00)	1.00 (1.00,2.00)	-0.35(-0.63,-0.07)	0.015
Renal subscore				
Day 1	0.00 (0.00,1.25)	0.00 (0.00,1.00)		
Day 3	0.00 (0.00,1.00)	0.00 (0.00,1.00)	-0.23(-0.55,0.08)	0.152
Day 7	0.00 (0.00,0.75)	0.00 (0.00,0.00) ^*^	-0.27(-0.53,-0.01)	0.043

Abbreviations: IL-6, Interleukin-6; BUN, bood urea nitrogen; Scr, serum creatinine; Lac, lactic acid

* Compared with Day 1, P (Bonferroni adjusted) < 0.05

^†^ Compared with Day 3, P (Bonferroni adjusted) < 0.05

Compared with the non-DEX group, the DEX group had no substantial advantage in pSOFA score during the 7-day observation period. Additionally, we analyzed the trajectories of the six sub-scores. In contrast to the non-DEX group, the DEX group showed significantly lower neurological and renal sub-scores at day 7, though there were no statistical differences in respiratory, coagulation, hepatic and cardiovascular sub-scores between the two groups. (Table 3)

### Effects of dexmedetomidine on outcomes

**Fig. 3:**
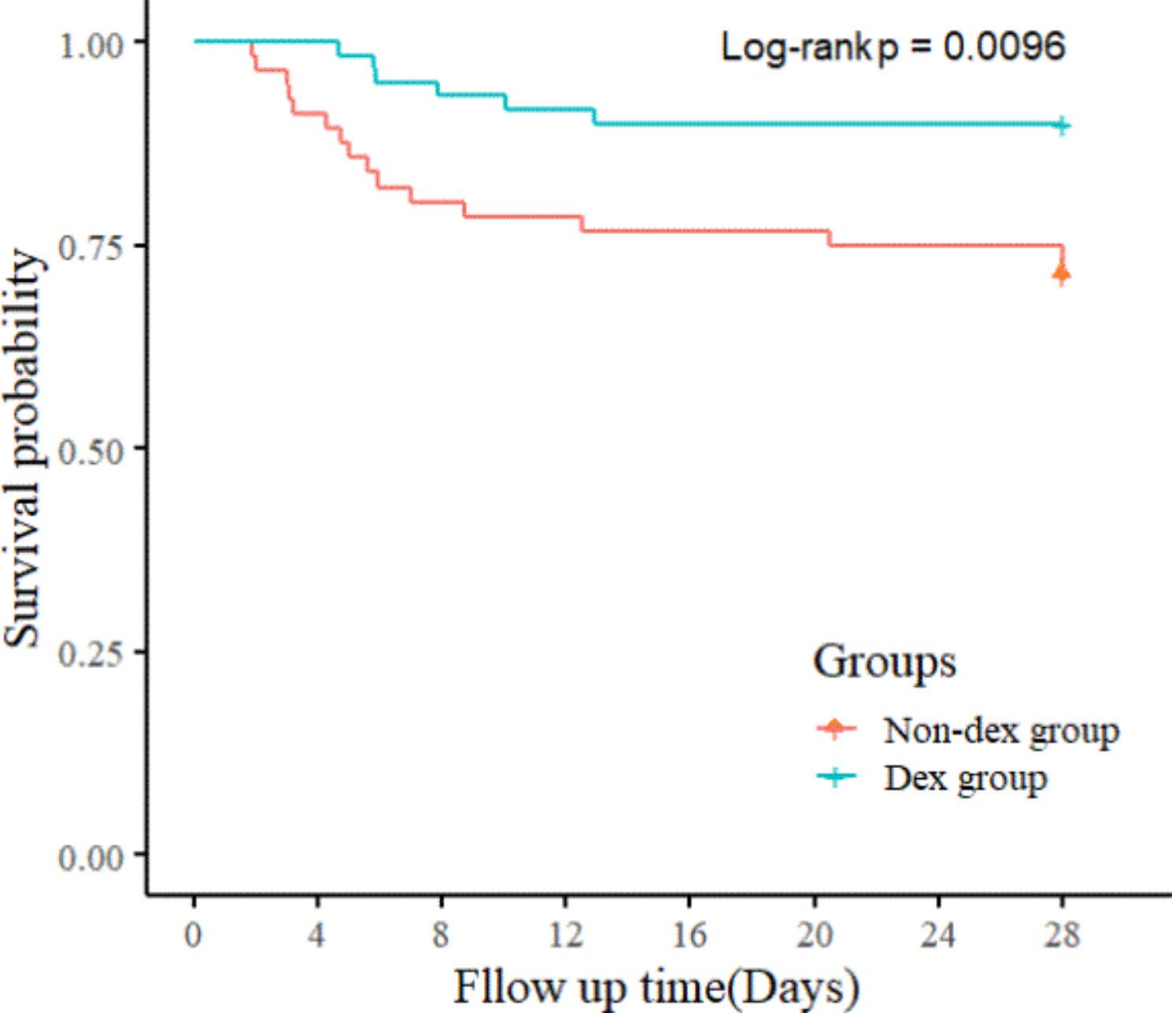
Kaplan-Meier curve showing probability of survival during the first 28 days of the two group, among mechanically ventilated pediatric patients with severe sepsis

After PSM, the 28-day mortality rate in the DEX group was significantly lower than that in the non-DEX group (9.84% vs. 26.23%, P = 0.008). By plotting the 28-day Kaplan-Meier cure, the survival rate of the DEX group was significantly higher than that of the non-DEX group (P = 0.0096) (Fig. 3). Although the incidence of AKI was lower in the DEX group than that in the non-DEX group, the difference was not statistically significant (9.84% vs. 19.67%, P = 0.063). There were no significant differences in ventilator-free, the length of PICU and hospital stay between the two groups. (Table 4)

**Table 4:** Comparison of the outcomes between the DEX group and non-DEX group

Outcomes	Non-DEX group	DEX group	HR/OR/Beta (95% CI)^*^	*P value* ^*^
**Primary outcome**				
28-day mortality	16 (26.23%)	6 (9.84%)	0.24 (0.08–0.69) ^†^	0.008
**Secondary outcomes**				
AKI (n [%])	12 (19.67%)	6 (9.84%)	0.27 (0.07–1.07) ^‡^	0.063
Ventilator-free days (median [IQR])	26.00 (21.00,28.00)	25.00 (21.00,28.00)	-0.65(-3.08,1.77)^§^	0.593
PICU stay, days (median [IQR])	12.07 (5.86,17.51)	14.34 (7.87,18.29)	0.68(-3.21,4.56)^§^	0.730
Hospital stay, days (median [IQR])	13.72 (6.90,25.01)	18.27 (12.75,24.85)	2.47(-2.24,7.17)^§^	0.301

Abbreviations: CI, confidence interval; HR, Hazards ratios

^*^ Corrected for baseline variables: age, weight, surgical patients, pSOFA-1d, No. of organs damaged, septic shock, use of vasoactive drugs, blood purification and fluid resuscitation

^†^ HR, estimated by cox proportional-hazards analysis

^‡^ Odds ratio (OR), estimated by logistic regression analysis

^§^ Beta, estimated by linear regression analysis

## Discussion

This study included 250 pediatric cases with severe sepsis and received mechanical ventilation, in which the 28-day overall mortality rate was 18.8%. After 1:1 PSM, DEX group showed more lower 28-day mortality than non-DEX group (9.84% vs. 26.23%, P = 0.008). During the 7-day observation period after PICU admission, although there was no statistical difference in the incidence of AKI between the two groups, the DEX group showed a statistically significant reduction in Scr level at day 3 and day 7 compared with the non-DEX group. Furthermore, in contrast to the non-DEX group, the DEX group showed significantly lower neurological and renal sub-scores at day 7, though there were no statistical differences in respiratory, coagulation, hepatic and cardiovascular sub-scores between the two groups.

A retrospective cohort study in Japan involving 50,671 adult patients who received mechanical ventilation for sepsis showed that the use of dexmedetomidine was associated with reduced all-cause 28-day mortality and a shorter duration of mechanical ventilation [19]. Likewise, Kawazoe Y et al. found that the 28-day mortality of patients with sepsis treated with dexmedetomidine decreased by 8%, though the difference was not statistically significant [20]. Sequentially, Kawazoe Y and his colleagues conducted a subgroup randomized controlled trial and then found that dexmedetomidine significantly reduced the in-hospital mortality in septic patients with Acute Physiology and Chronic Health Evaluation II (APACHE II) scores ≥ 23 [9]. In line with these previous studies in adults, our study found that, for pediatric severe sepsis with mechanical ventilation, dexmedetomidine could decrease the 28-day mortality by 16.39% while the duration of mechanical ventilation did not change significantly.

Moreover, we compared the change of renal function between using or not using dexmedetomidine. We found that the renal sub-score of pSOFA was significantly improved and the decrease of Scr level (at day 3, day 7) was significantly greater in the DEX group than that in the non-DEX group, which indicated dexmedetomidine had protective effect on kidney. However, there was no significant difference between these two groups in the incidence of AKI, which may because the sample size is not enough.

Dexmedetomidine also was reported having neuroprotective effects in ischemia/reperfusion [21]. Pandharipande et al. found that patients with sepsis who received dexmedetomidine had more time without brain dysfunction than those who took lorazepam [11]. Mei B et al. showed that dexmedetomidine reduced systemic inflammation, neuroinflammation, injury of BBB and cognitive dysfunction in septic mice [22]. The study by Tain M et al. suggested that the neuroprotective effect of dexmedetomidine on septic mice was achieved by correcting peripheral Th1/Th2/Th17 shift and reducing proinflammatory cytokines in the hippocampus [23]. In the present study, we found that the use of dexmedetomidine reduced the neurological subscore of pSOFA in children with severe sepsis over the 7-day observational period, further supporting the protective effects of dexmedetomidine.

As far as the potential mechanism, several studies have shown that, unlike other sedatives such as midazolam and propofol, dexmedetomidine as an α2 agonist may potentially modify inflammatory and immune pathways by a number of ways under acute inflammatory conditions [24]. Firstly, dexmedetomidine could alleviate the inflammatory reaction [24–28]. Some studies in animals have shown that dexmedetomidine could prevent sepsis-induced AKI by reducing the levels of inflammatory cytokines, such as tumor necrosis factor-alpha, monocyte chemotactic protein-1 and interleukin-6, and ameliorate renal dysfunction [29, 30]. Secondly, dexmedetomidine was reported to be able to activate the cholinergic anti-inflammatory pathway and then reduce sepsis-related lung injury in mice [31]. Furthermore, dexmedetomidine can alleviate the oxidative stress reaction and reduces apoptosis in the pathogenesis of sepsis [32–34]. In addition, dexmedetomidine was also found to be able to reduce the level of norepinephrine in the blood, resulting in an increase in renal blood flow and urinary output [35]. In general, dexmedetomidine was speculated to improve organ functions by multiple mechanisms, thereby reducing the mortality of sepsis.

In our study, over the 7-day observation period, the decrease of IL-6 level in DEX group was slightly more than that of non-DEX group (no statistical difference) ,meanwhile, the level of PCT showed the same trend. All these may support dexmedetomidine could reduce the inflammatory reaction.

Several limitations need to be acknowledged. First, due to a retrospective cohort study, the differences in baseline characteristics between groups may affect the results of the study. The PSM analysis could balance the baseline characteristics between the two groups. Therefore, the influence of confounding factors should be small. But some confounding factors, such as the timing of antimicrobial initiation, are still hard to avoid in retrospective study. Second, we investigated the effect of dexmedetomidine on mortality and organ function in mechanically ventilated children with sepsis, but the mechanism remains unclear which merits further study. Third, we included only mechanically ventilated children with sepsis. It remains unclear whether dexmedetomidine is effective for children without mechanical ventilation. Further research is needed.

## Conclusion

The results from this study indicate that the use of dexmedetomidine may improve the mortality of pediatric severe sepsis patients with mechanical ventilation, probably by a multi-organ protective effect. However, further studies are needed to verify the benefits and identify the specific mechanisms.

### Electronic supplementary material

Below is the link to the electronic supplementary material.

Supplementary Material 1

Supplementary Material 2

## Data Availability

The datasets supporting the conclusions of this article are included within the article. The underlying datasets are available from the corresponding author on reasonable request.
