# Supplementary material for: Dexmedetomidine improves the outcomes for pediatric severe sepsis with mechanical ventilation
Source: BMC Pediatr. 2023 Aug 18;23:406. doi: 10.1186/s12887-023-04232-6 (PMC10436587; doi:10.1186/s12887-023-04232-6)
Supplement: Supplementary file 1 — Supplementary Material 1 [file 12887_2023_4232_MOESM1_ESM.docx]

**Suppl Table 1. Usage dose and duration time of dexmedetomidine after PSM**.

|  | DEX group | Non-DEX group |
| --- | --- | --- |
| N | 61 | 61 |
| Dose, median (IQR), mcg/kg | 88.10 (0.56, 310.02) | - |
| Duration time, median (IQR), hr | 173.25 (45.50, 257.00) | - |
