# Supplementary material for: Dexmedetomidine improves the outcomes for pediatric severe sepsis with mechanical ventilation
Source: BMC Pediatr. 2023 Aug 18;23:406. doi: 10.1186/s12887-023-04232-6 (PMC10436587; doi:10.1186/s12887-023-04232-6)
Supplement: Supplementary file 2 — Supplementary Material 2 [file 12887_2023_4232_MOESM2_ESM.docx]

**Suppl Table 2. Pediatric Sequential Organ Failure Assessment Score**

| Variables | Score^a^ | | | | |
| --- | --- | --- | --- | --- | --- |
|  | **0** | **1** | **2** | **3** | **4** |
| Respiratory | | | | | |
| PaO_2_:FiO_2_ ^b^  or  SpO_2_:FiO_2_ ^c^ | ≥400 | 300-399 | 200-299 | 100-199 With respiratory support | ＜100 With respiratory support |
|  | ≥292 | 264-291 | 221-264 | 148-220 With respiratory support | ＜148 With respiratory support |
| Coagulation | | | | | |
| Platelet count, ×10^3^ /μL | ≥150 | 100-149 | 50-99 | 20-49 | ＜20 |
| Hepatic |  |  |  |  |  |
| Bilirubin, mg/dL | ＜1.2 | 1.2-1.9 | 2.0-5.9 | 6.0-11.9 | ＞12 |
| Cardiovascular |  |  |  |  |  |
| MAP by age group or vasoactive infusion, mm Hg or μg/kg/min^d^ |  | | | | |
| ＜1 mo | ≥46 | <46 | Dopamine hydrochloride ≤5 or dobutamine hydrochloride (any) | Dopamine hydrochloride >5 or epinephrine ≤0.1 or norepinephrine bitartrate ≤0.1 | Dopamine hydrochloride >15 or epinephrine >0.1 or norepinephrine bitartrate >0.1 |
| 1-11 mo | ≥55 | <55 |  |  |  |
| 12-23 mo | ≥60 | <60 |  |  |  |
| 24-59 mo | ≥62 | <62 |  |  |  |
| 60-143 mo | ≥65 | <65 |  |  |  |
| 144-216 mo | ≥67 | <67 |  |  |  |
| >216mo^e^ | ≥70 | <70 |  |  |  |
| Neurologic |  | | | | |
| Glasgow Coma Score^f^ | 15 | 13-14 | 10-12 | 6-9 | <6 |
| Renal |  | | | | |
| Creatinine by age group, mg/dL |  | | | | |
| ＜1 mo | <0.8 | 0.8-0.9 | 1.0-1.1 | 1.2-1.5 | ≥1.6 |
| 1-11 mo | <0.3 | 0.3-0.4 | 0.5-0.7 | 0.8-1.1 | ≥1.2 |
| 12-23 mo | <0.4 | 0.4-0.5 | 0.6-1.0 | 1.1-1.4 | ≥1.5 |
| 24-59 mo | <0.6 | 0.6-0.8 | 0.9-1.5 | 1.6-2.2 | ≥2.3 |
| 60-143 mo | <0.7 | 0.7-1.0 | 1.1-1.7 | 1.8-2.5 | ≥2.6 |
| 144-216 mo | <1.0 | 1.0-1.6 | 1.7-2.8 | 2.9-4.1 | ≥4.2 |
| >216mo^e^ | <1.2 | 1.2-1.9 | 2.0-3.4 | 3.5-4.9 | ≥5 |

Abbreviations: FiO_2_, fraction of inspired oxygen; MAP, mean arterial pressure; pSOFA, pediatric Sequential Organ Failure Assessment; SpO_2_, peripheral oxygen saturation. SI conversion factors: To convert bilirubin to micromoles per liter, multiply by 17.104; creatinine to micromoles per liter, multiply by 88.4; and platelet count to ×10^9^ /L, multiply by 1.

^a^ The pSOFA score was calculated for every 24-hour period. The worst value for every variable in each 24-hour period was used to calculate the subscore for each of the 6 organ systems. If a variable was not recorded in a given 24-hour period, it was assumed to be normal and a score of 0 was used. Daily pSOFA score was the sum of the 6 subscores (range, 0-24 points; higher scores indicate a worse outcome).

^b^ PaO_2_ was measured in millimeters of mercury.

^c^ Only SpO_2_ measurements of 97% or lower were used in the calculation.

^d^ MAP (measured in millimeters of mercury) was used for scores 0 and 1; vasoactive infusion (measured in micrograms per kiligram per minute), for scores 2 to 4. Maximum continuous vasoactive infusion was administered for at least 1 hour.

^e^ Cutoffs for patients older than 18 years (216 months) were identical to the original SOFA score.

^f^ Glasgow Coma Scale was calculated using the pediatric scale.

Matics TJ, Sanchez-Pinto LN. Adaptation and Validation of a Pediatric Sequential Organ Failure Assessment Score and Evaluation of the Sepsis-3 Definitions in Critically Ill Children. JAMA Pediatr. 2017;171(10):e172352.
